# Notes and News

**Published:** 1893-07-22

**Authors:** 


### Notes and News.

Collected and Communicated.

The annual inspection of the V.M.S C. took place at Chelsea Barracks on Saturday, the 15th inst., and on August 5th the corps is going to Aldershot for a week's training under canvas.

That remarkable French calculator, Inaudi, has an English rival in Mr. Henry Crawford, a journalist, at Harrow. He gave an exhibition last week of his remarkable abilities to an audience in London. His powers of memory and of rapid calculation are alike remarkable.

Already 3,300 fatal cases of cholera have occurred in Podolia. At Toulon four out of the five cases reported in one day proved fatal. From Alexandria over eighty pilgrims have been admitted at the Quarantine Station suffering from the disease. English cholera is reported to be very prevalent in Manchester.

The annual meeting of the Shipwrecked Mariners' Benevolent Society was held last week. At the meeting it was announced that the Duke of York had consented to become a patron. The Society expended £22,039 in affording assistance last year. A very large number of mariners subscribe to the funds of this beneficent Society.

An object lesson in the carelessness of the lower classes in spreading infectious disease was recently given. A patient is stated to have escaped the vigilance of the Guy's Hospital authorities and returned to his home where dress-making was carried on. The fine of forty shillings inflicted for the offence appears hardly likely to prove a severe warning.

The Duchess of Fife presented prizes last week to the pupils of the Association for the Oral Instruction of the Deaf and Dumb. The system of instruction pursued by the Association has proved most successful, and the institution provides training for a number of teachers also. Funds are much needed, and as the undertaking is well worthy of support, we trust these will be forthcoming.

The sum collected for the building fund of the new wing at St. Mary's Hospital is £20,000, not £11,000 as generally stated.

A source of danger has been revealed recently in the person of a doctor's boy, who left medicine at the wrong house. This resulted in the death of a baby to whom the medicine for an adult was administered, the mistake being discovered too late.

The United Hospitals' Challenge Cup was shot for at the N.R.A. meeting at Bisley on Thursday, the 12th, St. Thomas's winning the cup with a score of 364, Guy's (327), St. Mary's (317), and Charing Cross (224) following in the order named.

The Volunteer Medical Staff Corps turned out for ambulance work on the occasion of the Royal wedding to the number of 150 officers and men, under the command of Surgeon-Lieut.-Colonel Platt and Surgeon-Captains Hayes, Matthews, and Raw. They were able to render first aid to about 500 cases, the most serious being that of a woman (who afterwards died in St. George's Hospital), a man who sustained a fracture of the radius, and the Marquis of Tullibardine, of the Royal Horse Guards, who was thrown from his horse in the courtyard of Marlborough House.

King's College Hospital, an institution which has done splendid work, and which is now ably administered, especially in the nursing department, has spent the whole of its available capital, and owes in addition £6,000 to its bankers. The Rev. Henry Wace, D.D., and Mr. Richard Twining, the chairman and treasurer respectively, make an urgent appeal for the sum of £50,000, to constitute a maintenance fund for seven years, and they certainly ought to receive at least this sum within a month, seeing that King's College is the hospital of the Bench and the Bar, and the home of Lister, the most illustrious of living surgeons, whose work has immeasurably lengthened the lives of thousands of the present generation, and of millions of those who have yet to come.

The committee of the Meath Home for Epileptics are fortunate in having secured the services of a most excellent Lady Superintendent, Miss Anderson. Miss Anderson is a thoroughly trained nurse, an excellent manager, and an enthusiastic worker. The Health Home is a happy instance of an institution where committee and management work together cordially with the best interests of the undertaking in view. Delightful little services are held in the Home, in which all join heartily, and one patient on the day we were present presided at the piano and played the hymns excellently. It is intended as soon as it is feasible to convert a large barn, which forms part of the premises, into an excellent chapel. A very complete laundry is now nearly finished. The establishment will eventually be thoroughly self-contained, and leave little to wish for. The patients are thoroughly happy and comfortable. No pains are spared to improve their sad condition. An excellent medical man joins the efforts of Matron and Committee in this work. Useful occupation, as well as amusement, is provided; and really charming Japanese bead and bamboo blinds, as well as admirable plain work, are produced by the inmates of the Home, who are much helped by orders from without. Encouragement of all kinds will not be wanting from those who have had the privilege of acquaintance with the work of this veritable haven for the afflicted.

